# *From ‘spectating’ to ‘spect-acting’*: medical students’ lived experiences of online Forum Theatre training in consulting with domestic abuse victims

**DOI:** 10.1186/s41077-022-00208-1

**Published:** 2022-04-15

**Authors:** Daire McGrath, Gerard J. Gormley, Helen Reid, Paul Murphy

**Affiliations:** 1 Centre for Medical Education, School of Medicine, Dentistry and Biomedical Sciences, Queen’s University Belfast, Belfast, Northern Ireland BT785BN; 2 Drama Studies, School of Arts, English and Languages, Queen’s University Belfast, Belfast, Northern Ireland

**Keywords:** Domestic abuse, Forum Theatre, General practitioners (GPs), Simulation, Medical student

## Abstract

**Introduction:**

Health care professionals, including general practitioners, have an important role in the care of those affected by domestic abuse. Therefore, it is important that healthcare professionals are adequately trained in recognising features of domestic abuse and supporting victims in disclosure. Founded by Augusto Boal, Forum Theatre is a drama methodology that can permit an experiential and immersive learning experience; lending itself well to a subject matter of oppressed individuals. In this study we aimed to gain a deep understanding of medical students’ lived experiences of training in consulting with individuals who experienced domestic abuse using an online format of forum theatre.

**Methods:**

A multidisciplinary team developed an online forum theatre training exercise, which involved a simulated consultation between a general practitioner and domestic abuse victim. Our qualitative approach used hermeneutic phenomenology to explore the participants’ lived experiences of this training. Following the online forum theatre experience, we analysed 11 participant interviews using template analysis to structure the phenomenological interpretation.

**Results:**

We developed five themes through our analytical process: 1) ‘Almost being there…but not quite’: the realistic experience of forum theatre; 2) ‘Taken on an emotional journey’ 3) ‘Opening and controlling a privileged space’; 4) ‘Small things matter…’: cultivating and maintaining rapport and 5) Critically reflecting on future professional self.

**Discussion:**

This study offers fine-grained insights into medical students’ experiences of an online immersive forum theatre training exercise in consulting with individuals who have been affected by domestic abuse. Online forum theatre has the potential to provide a simulated and meaningful approach to train medical students about domestic abuse.

By providing students with a unique opportunity to step into a General Practitioner’s shoes in a domestic abuse consultation, students can practise how they manage a consultation with an impacted individual through a safe, guided, and experiential approach.

**Supplementary Information:**

The online version contains supplementary material available at 10.1186/s41077-022-00208-1.

## Introduction

Domestic abuse (DA) is a prevalent and harsh reality in our societies. According to the Office for National Statistics, over 2.4 million adults in England and Wales experienced DA in 2019 [1]. Globally, 27% of women aged between 15 and 49 years who have been in a relationship have experienced physical and/or sexual violence by their intimate partner [2]. Moreover, many more incidents of DA go unreported for a number of reasons including fear of retaliation, financial dependency, emotional attachment, embarrassment and social stigma [3]. During the COVID-19 pandemic, there has been a marked increase in DA, attributable to the abused having to stay at home with their abuser for longer periods of time. Refuge (a UK DA charity) reported a 700% increase in online traffic to its national website [4].

### Domestic abuse: the role of healthcare professionals

Healthcare professionals (HCPs) play a vital role in the care of individuals who are subjected to DA. The abused have more healthcare interactions and receive more prescriptions on average in comparison with the general public [5]. Whilst we know that many individuals may be fearful around abuse disclosure, it is important to note that many would like their doctors to be proactive in asking about DA. It is therefore important that HCPs are adequately trained to recognise features of DA, how to assist the abused in disclosing and provide appropriate care upon disclosure.

Following a successful 2011 randomised controlled trial [6], the ‘Identify and Referral to Improve Safety’ scheme was implemented in general practices across the UK [7]. This has led to an increased number of DA service referrals in the UK [8], but there is still room for improved training for general practitioners (GPs), medical students, and other HCPs. Specifically, in this paper, we will focus on DA teaching for medical students. A cross-sectional study carried out across UK medical schools found that DA training only lasted between 0 and 2 h in 52% of schools [9].

A range of teaching methods are used in extant DA training for HCPs and students. Many of these forms of learning focus more on the *intellectual* rather than the *experiential* aspects of providing professional care for the abused [10]. Intellectual forms of learning such as reading materials and group discussions can fall short of best preparing HCPs and students to provide care in real clinical practice. There can be a disconnect between what is *learnt* and how they *act* in practice. Though HCP students may encounter the abused during clinical placements, such opportunities to learn are inconsistent and do not facilitate students to directly assist a patient in disclosure about their abuse.

Experiential forms of learning such as simulation can create opportunities to gain a more embodied and behavioural learning experience. Embodied learning refers to pedagogical methods which concentrate on holistic aspects of learning and that signify the importance of the body and feelings [11]. There has been a recent trend towards online forms of experiential learning concomitant with a shift towards virtual provision due to COVID-19. As with all forms of learning, there needs to be alignment between desired learning outcomes and the method of teaching. Whilst acknowledging the limitations associated with online learning, there are also affordances including scalability, accessibility and environmental benefits associated with reduction in travel to simulation centres. Of the many forms of experiential learning, *Forum Theatre* (FT) lends itself to the subject matter of DA.

### Forum Theatre: giving voice to the oppressed

The methodology of FT, as an aspect of *Theatre of the Oppressed*, was founded by Brazilian drama theorist, practitioner and social activist Augusto Boal. The theory was presented in Boal’s book, the *Theatre of the Oppressed*, which was heavily influenced by Paulo Freire’s book, the *Pedagogy of the Oppressed* [12]. The main concept of FT within the *Theatre of the Oppressed* is to break down barriers which often exist in theatre, specifically between the actors and the audience — spectators — who behold the action on stage. A significant innovation is that during a scene, the spectators can intervene directly in the dramatic action as it is manifest in a sequence of activities. Initially, the scene is performed by the actors. The scene is then repeated, and spectators are invited to take the place of an actor or guide the actor and decide what action to take, thus helping to change the outcome of the scene. The spectators effectively become actors, a change of status encapsulated in the neologism ‘spectactor’. Other actors must react instantly to the new scenario.

The interactivity of FT evokes a desire to practise the act that has been improvised. With recent rapid expansion of virtual health consultations, it could be argued that an online adaptation of FT may provide medical students opportunity to learn how to authentically consult with and support an individual who has experienced DA. Given the importance of making ‘in the moment’ decisions during such forms of experiential learning, it would be important to know what learners actually experience and how this may impact their professional development. In this study, we aimed to gain a deep understanding of medical students’ lived experiences of training in consulting with those who experienced DA using an online version of FT.

## Methods

### Conceptual orientation of study

Our research aim required a methodological approach that could provide in-depth insights to lived experiences of an online FT experience. Phenomenology is well-established in health professions education research as a means of elucidating nuanced insights about individuals’ lived experiences [13]. In the *descriptive* tradition of phenomenology, the focus is describing how a phenomenon appears in an individual’s consciousness. The aim of *interpretative* forms of phenomenology, such as hermeneutics, is to understand how lived experiences are constructed and interpreted whilst acknowledging that researchers bring their own experiences to the analytic process. Hermeneutic phenomenology was therefore a good conceptual fit for our study.

### Research ethics

Ethical approval (MHLS 20_148) was provided by the Faculty of Medicine, Health and Life Sciences Research Ethics Committee, Queen’s University Belfast (QUB). Written informed consent was obtained from all participants. We complied with the COnsolidated criteria for REporting Qualitative research (COREQ) checklist throughout our study [14].

### Study setting

Our study was set in the Centre for Medical Education at QUB, Northern Ireland, where the medical degree programme follows a 5-year integrated curricular model. Students have clinical placements, including GP, across their 5 years of training.

### Subject recruitment and selection

Years 3 and 4 medical students (*n* = 544) were invited to participate in the study by email. We aimed to have a balanced gender composition among participants, using a maximal variation sampling approach (a matrix sampling grid across reported student genders and on a first-come, first-served basis). Sampling numbers in phenomenologically based research are generally much smaller compared to other methodologies permitting in-depth analysis without being overwhelmed by data. We therefore aimed to recruit 8–12 participants to gain deeper insights into participants’ lived experiences compared to the broader insights we might have gained from a larger sample [15]. Overall, 16 students indicated interest in taking part in the study, with 11 students being recruited for the study.

### Online forum theatre: a description

A multidisciplinary team developed an online FT learning activity. Grounded in the principles of FT, the primary aim was to provide medical students with a realistic experience of a GP consulting with an individual who has experienced DA. Specifically, we focused on DA detection, supporting the individual to consider disclosure, and ensuring safety.

The team comprised an academic GP (HR), simulationist and academic in drama (PM), simulationist and GP (GG) and a medical student (DMcG). We drew upon real-life experiences of DA provided by a professional involved in the care of individuals who have been subjected to DA (SMcM). In a confidential manner, SMcM shared with the research team a range of first-hand accounts of individuals who have been subjected to DA. Such important lived experiences were used to inform the development of our FT activity. The FT activity was storyboarded and developed iteratively, with the input from SMcM who drew upon her experiences of caring for people who have been subjected to DA; full description contained in Additional file 1. Senior drama students and HR rehearsed and refined the FT piece. Attention was paid to the actors’ characterisation, dress, scenes, props and make-up (for example the bruise on the patient’s neck). Microsoft (MS) Teams was used as the online interactive platform for this activity. All members of the research team, and SMcM, approved the final version of the FT activity.

Consenting participants experienced the FT in groups of three to four learners. We carried out three separate FT sessions lasting approximately 1 h. All members of the research team were present in these FT sessions. A distress protocol was devised in the event any participant, or drama student, experienced any distress during the FT activity. At no point during, or after, the study — did any individual declare they had any distress.

### Data collection

**Fig. 1:**
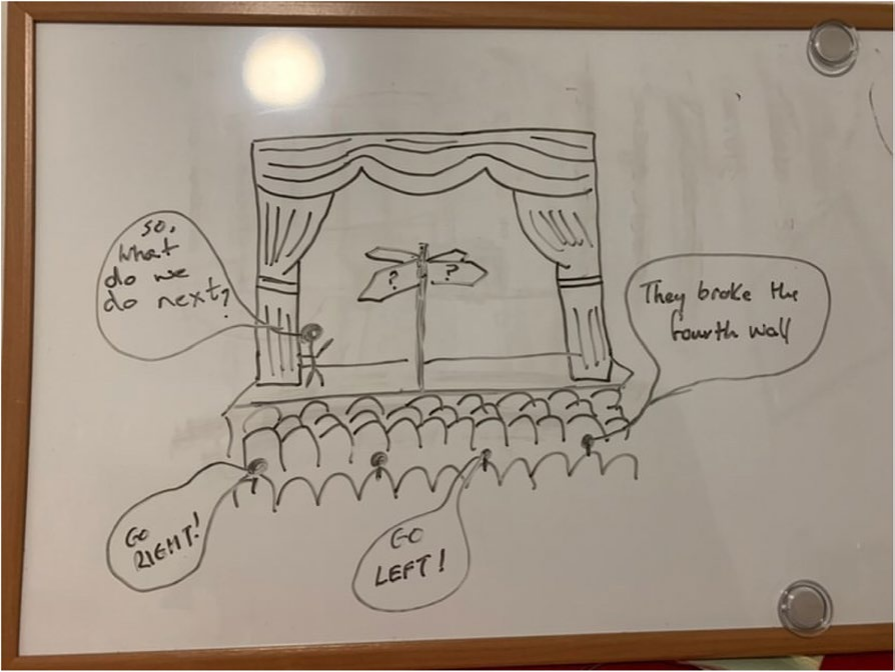
Illustration of a qualitative interview elicitation rich picture by “Phillip”*. *All names included in this paper are pseudonyms

**Fig. 2:**
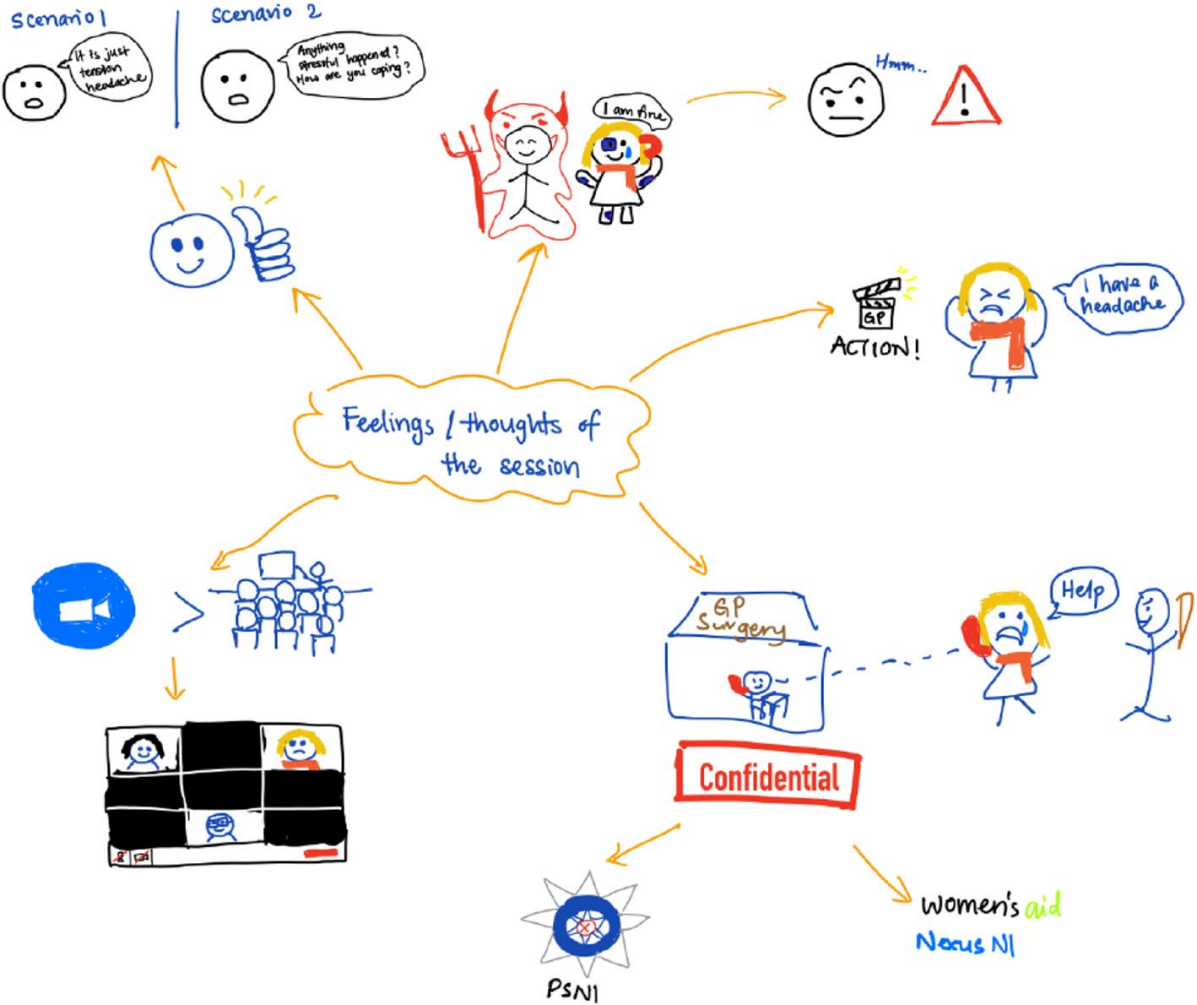
Illustration of a qualitative interview elicitation rich picture by “Amelia”

In the days following the online FT, participants took part in one-to-one qualitative interviews conducted via MS Teams, by DMcG (trained in qualitative research interview techniques and having no relationship with participants). In keeping with phenomenological research, interviews were minimally structured to keep them grounded in participants’ experiences. A brief question guide was used to initiate the interviews. Rich pictures were used as an interview elicitation technique to enrich participants’ expression of their experiences (see Figs. 1 and 2 for an example of a rich picture) [16]. Prior to the interview, participants were asked to draw a picture that symbolised their experience of the FT activity.

Research interviews were recorded using digital dictaphones, transcribed verbatim, checked for accuracy and anonymised using pseudonyms.

### Analysis

A template analysis approach was used given its epistemological fit with hermeneutic phenomenology [17]. Template analysis provides a structured approach to analysing participants’ experiences, researchers’ interpretations, and how the data contributes to overall understanding of how medical students experienced the FT activity. Interview transcripts were the focus of analysis. Rich pictures were not directly analysed.

Tentative a priori codes were identified following literature review in line with our research aim. Initially, these codes were applied to one transcript in order to identify relevant experiences. Following this, preliminary themes were identified by refining a priori codes and organising themes into clusters, thus generating an initial template. Themes were progressively refined by applying the initial template to remaining transcripts. Finally, all transcripts were coded against the final template. Analysis concluded once all researchers agreed that sufficiency of data in terms of thick and rich description had been achieved. Member checking was conducted to seek respondent validation. Throughout analysis, the research team was reflexive through a process of discussion, review and writing.

## Results

Eleven participants (9 female and 2 male) were interviewed, generating 373 min of data. Five themes were derived through our analytical process:

1. ‘Almost being there…but not quite’: the realistic experience of Forum Theatre
2. ‘Taken on an emotional journey’
3. ‘Opening and controlling a privileged space’
4. ‘Small things matter…’: cultivating and maintaining rapport
5. Critically reflecting on future professional self

### ‘Almost being there…but not quite’: the realistic experience of Forum Theatre

Despite the simulated nature of the online FT, participants experienced a sense of realism about the DA consultation. Though participants were aware this was a ‘created’ enactment, they were drawn into the situation and experienced a heightened sense of realism.

*….you actually almost had to just remember that this isn’t real, that these people are just actors…* (Yasmin)

As medical students, they were unlikely to have experienced such sensitive consultations in their actual clinical training. However, the FT piece did trigger an imagined sense of what such a consultation might be like and an ability to consider perspectives from both the GP, the abused and the abuser.

*It felt like it was a real consultation and that could have been a real patient you were watching.* (Charlie)

A number of factors appeared to shape the realism. The authenticity of the ‘story’ helped make the FT a believable experience for participants. Aside from their props, clothing and make-up, the actors’ ability to express their respective characters through dialogue and gestures was an important factor.

The online nature of the FT piece did not appear to detract from the realism experienced by participants. With an increasing trend towards remote consultations (especially given the COVID-19 pandemic), participants appeared to be more accepting of this mode of engagement.

*It was good to see a consultation happening over video call, because that is becoming so much more common.* (Eimear)

### ‘Taken on an emotional journey’

The phenomena of the online FT activity evoked a range of emotional experiences in participants. Whilst the emotions experienced by participants could vary, patterns were nonetheless discernible as the FT piece unfolded. The emotions that were intentionally portrayed by the actors were, to a certain degree, experienced by participants.

*When Jane started crying, I wanted to cry too.* (Mia)

The emotional experience seemed to also be driven by the degree of perceived responsibility that participants experienced.

*A feeling of anticipation… like now I need to act, like she’s told me this, now I need to do something about it and get her out of there. Even though I’m not the GP, that’s how it makes you feel, the Forum Theatre, like you feel responsible for her.* (Eimear)

More negative emotions such as frustration and discomfort were evoked in response to the suffering of the abused individuals in the FT. Negative emotions including anger and anxiety were also experienced in response to the abuser.

*When we watched the first part when the husband phoned Jane, saying ‘don’t go here, don’t go there, just wait for me’, that made me feel anxious.* (Eimear)

As the FT piece progressed, negative emotional experiences were often replaced by more positive emotions such as relief and hope. The more positive emotions mirrored the unfolding interaction between the GP and the abused individual, as the GP facilitated disclosure and provided support. Given that the participants had a ‘hand’ in directing the course of the GP’s actions, this appeared to reinforce these positive emotions.

*When we were able to change the course of the consultation, I felt like a relief that it was sorted out.* (Eimear)

Participants, whilst experiencing different degrees of emotional intensity, did not feel distressed by these experiences.

### ‘Opening and controlling a privileged space’

There was a uniqueness to what participants experienced in the FT piece. They appreciated the FT as a privileged opportunity to engage with a sensitive consultation as it unfolded in front of them. The ability to pause the consultation was a powerful experience for participants.

*I feel like if you’re just trying to catch on to this at placement to learn it, you know, not everyone will see it. Also, it’s quite likely that the patient will say, can we not have a medical student here, and then you won’t get that learning experience from it.* (Alex)

This event provided them opportunities, while feeling a sense of being ‘in the moment’ of the simulated consultation, to think, reflect and share their thoughts with others, which students valued.

*being able to practise, and sort of pause, and stop, and think… to basically get a dry run of that in a safe environment.* (Leah)

This simulation allowed participants to share their thoughts and not only consider next steps but importantly find the words to convey them.

*It’s good to have practice when it’s a simulation rather than a patient in front of you and scrambling to try and figure out what you should say and do.* (Charlie)

### ‘Small things matter…’: cultivating and maintaining rapport

As participants engaged with the FT piece, they experienced a strong desire to develop a rapport with the vulnerable individual whose plight they witnessed. Aside from the medical dimension (in this consultation a tension headache), there was a realisation of the importance of what might be considered ‘less important details’ such as tone of voice.

*It was a good way to show how small changes in a consultation have positive effects.* (Eimear)

Participants experienced the importance of considering the wider consultation and ‘joining the dots’ between the various individual features that alone might not arouse suspicion of DA. In this process, participants experienced the need to be more proactive. In so doing, they understood the priority to display empathy with the patient.

*Even changing the tone of your voice or the way you are asking a question makes someone feel more open to talking to you.* (Eimear)

Creating a safe space to explore key issues with a patient was important, and displaying genuineness in their situation was fundamental. The lack of such supportive conditions could be a ‘make or break’ point in the process of a patient disclosing abuse.

### Critically reflecting on future professional self

Through the FT, participants experienced a greater awareness of the role of a doctor when interacting with patients who have been subjected to DA. More than just imagining what this experience would be like, the FT enabled consideration of actions they could take to better consult with and support an individual to disclose abuse.

*If a patient is hesitant, maybe ask them about their hesitance instead of just going past it because they might want you to ask them something.* (Maria)

Participants experienced a sense of conditions to enable disclosure, for example: taking time with a patient, considering that patients may be concealing their abuse, considering consultations holistically, providing a supportive environment, being proactive, and making every interaction count.

*I just need to be more aware… not to just focus on the straight history taking and examination, but also to try and pick up more on how they look, what sort of emotions does it look like they’re going through at the moment, and do they look uncomfortable, are they nervous, do they look like they’re trying to hide something, or that they just want to get out of there as soon as possible.* (Leah)

## Discussion

Online FT involving consultation with an individual who had experienced DA provided a unique and embodied simulated experience for medical students. Although difficult to replicate what a GP would experience, FT provided medical students a realistic opportunity to experience this scenario. The present study allowed students to experience the emotions evoked by a sensitive consultation in a controlled and guided manner. The online FT afforded medical students a unique opportunity which they may not experience through clinical placements. This simulated consultation instilled the concept that ‘small things matter’, enabled reflection on their own learning and skill set, and the importance of self-reflection in their future careers.

Our results highlight the impact of the FT on the participants. By providing a realistic simulated environment for learning about a DA consultation, students were able to reflect on their skill sets as future doctors. The sense of realism appeared to stimulate engagement and a sense of empathy from participants, in keeping with other studies showing that simulation improves empathy in medical students [18, 19]. This FT session was carried out online. Our initial assumption was that this might diminish the realism experienced by participants in comparison with an in-person session: this was not the case. At the time of writing, a large proportion of GP consultations are being carried out over the phone or online [20]; thus, participants thought that the online format paralleled real-world practice.

The online FT had an emotive effect on participants, mirroring the abused’s emotions and potentially stemming from the participants’ sense of responsibility in the simulated role of a GP. By experiencing this type of consultation from an emotional standpoint, students may develop an understanding of how it feels to be a part of a sensitive consultation. This may help their future selves develop empathy for the abused and control their own feelings, though future research would be required to determine if this is the case, particularly in a sustained fashion. This is important as literature suggests that educational interventions can maintain and improve empathy in undergraduate medical students [21].

The unique experience provided by FT allowed students to experience a simulated DA consultation scenario, which they would not experience within normal clinical training. Medical students were afforded a safe place to step into a GP’s shoes and direct how the consultation unfolded. The online FT simulation may help students to improve their consultation skills, including observational awareness of a patient’s body language and signs of physical abuse. FT simulation may also help students to develop listening skills, by being able to see the outcomes of their line of questioning, and then adapting it to see what works best.

Another dimension which added to the learning experience provided by online FT was the concept of ‘small things matter’. This helped to improve the awareness of participants to finer details such as gestures and providing a safe, comfortable environment for the abused which make a huge difference towards the overall consultation. Ultimately, this has potential to help medical students treat patients as individuals, which is an important trait in a caring doctor [22].

This learning experience enabled participants to learn actively and also encouraged them to think critically about their future career as HCPs. Medical students recognised the importance of self-reflection to their future careers and gained appreciation of what they knew and crucially what they did not know. The FT scenario also provided students with information about handling DA, including how to support the abused during and after disclosure and where to direct the abused. According to the General Medical Council, which is responsible for medical practitioners’ registration in the UK, there are four main principles to adult safeguarding for doctors: protection, empowerment, proportionality and partnership [23]. Medical students should strive to meet these standards when undergoing DA training.

### Implications for practice

Forum theatre has been used previously in medical student teaching to explore professionalism [24]. Our results indicate that FT is a putatively novel way to teach medical students about DA. If FT teaching for DA was included in medical schools’ curricula, then it may provide students with enhanced preparation for dealing with DA scenarios. However, there is a still more research required in this area, for example studying the impact of FT teaching for DA in participants at different stages of training and in other institutions. It may be useful to study a range of different DA scenarios, as this study only looked at one typical scenario. Moreover, the role of FT could be applied to a range of other subjects including addiction, child abuse, breaking bad news and other challenging conversations. Furthermore, it may also be interesting to introduce FT as a learning resource for qualified HCPs as well as students.

### Strengths and limitations

The findings in this study indicate that FT can be a novel teaching method around DA. This study was exploratory and achieved in-depth insights into FT use for DA teaching, providing a starting point for further research. However, findings should be considered under certain limitations. The main aim of our study was to gain a nuanced understanding of medical students’ lived experiences of training in consulting with those who experienced DA using an online version of FT. Reflexively, we acknowledge that we drew upon relevant expertise, including drama studies, to devise this study. Whilst we believe that such expertise provided us with deeper insights to participants’ findings, and skill set to develop the online FT piece, such expertise may not be readily available in all institutions, and therefore, findings may be less transferable. Now that we have gained a deep insight to learners’ experiences of such a learning activity, a feasibility study would help to explore how readily this may be transferable to others, especially with no experience in drama. Moreover, such a feasibility study would provide important information about the practical elements of implementing such an online FT learning activity, with a particular focus on the degree of expertise required to deliver such an activity and monitoring for unintended consequences. Findings from such a feasibility study could inform a larger trial of the utility of FT in the training of medical students and other HCPs.

Whilst we aimed for a gender balance in participants recruited for this study, we were unable to achieve an even balance. Whilst we intended not to specifically explore gender differences in the experience of this online FT learning activity, we acknowledge that having more female participants may well have had some bearing on our findings. Specifically, exploring the impact of gender of participants in an online FT learning activity would be worthy of future research.

As this study looked specifically at third- and fourth-year medical students, the results may not be transferrable to other medical students and HCPs. Furthermore, this study focused on a DA scenario involving a male perpetrator and a female victim. Although this is the most common DA scenario, it is important to acknowledge that there are other potential DA scenarios, and these may need to be handled differently. This study focused solely on the short-term impact of FT; therefore, it may be useful to carry out research into the long-term learning impact of FT.

## Conclusion

Teaching medical students about DA is an important aspect of training future doctors, and to this end, online FT provides a novel-simulated approach to learning. Forum theatre teaches medical students vital skills which are important in a DA consultation, such as awareness of body language, active listening and empathy, all of which are important skills needed to help support a DA victim during and after disclosure. The use of FT simulation to teach students about DA has the potential to enhance their ability in sensitively interacting and providing care for this important group of individuals. Further research is required to determine how such a FT learning activity could be implemented in other institutions and explore the impact of such an activity, particularly on learners’ empathic skills and unintended consequences.

## Supplementary Information

**Additional file 1.** Table showing a description of our FT piece regarding a victim of DA consulting with their GP.

**Additional file 2.** Interview Schedule.

## Data Availability

The datasets generated and/or analysed during the current study are not publicly available as digital files were deleted following transcription in keeping with data protection/ethical approval; however, anonymised transcripts are available on request from the author.

## Abbreviations

DA: Domestic abuse
HCPs: Healthcare professionals
GP: General practitioner
FT: Forum Theatre
