# Supplementary material for: From ‘spectating’ to ‘spect-acting’: medical students’ lived experiences of online Forum Theatre training in consulting with domestic abuse victims
Source: Adv Simul (Lond). 2022 Apr 15;7:11. doi: 10.1186/s41077-022-00208-1 (PMC9012059; doi:10.1186/s41077-022-00208-1)
Supplement: Supplementary file 1 — Additional file 1. Table showing a description of our FT piece regarding a victim of DA consulting with their GP. [file 41077_2022_208_MOESM1_ESM.pdf]

Additional file 1

Additional file 1; Table showing a description of our FT piece regarding a victim of DA consulting with their GP

1) **Briefing exercise**: Participants were briefed by identifying the main aim of the activity, clarification of the technical aspects, how to intervene in the FT piece and measures to ensure psychological safety. A short video was shown to participants that provided context about key issues regarding DA and the important impact that doctors can have in caring for such individuals.
2) **Prologue:** Following up a warm-up exercise, the facilitator ('Joker' is the term used in Boal's original formulation) introduced the main characters of the performance
    a. **Jane Millar** (fictitious name): Jane is a 26-year-old who lives at home with her partner Derek. They have no children. Derek is very controlling of Jane and tracks her whereabouts (even to the extent of using webcams at home). Jane has been experiencing escalating verbal and physical abuse. Derek has told Jane that if she 'tells anyone' he will 'kill himself'. Jane really wants to leave this relationship – however she feels trapped.
    b. **Derek Johnston** (fictitious name): Derek is a 30-year-old company director and travels a lot with work. He is very possessive of Jane and monitors all her actions. To the 'outside world' he is a 'nice guy' which is far from the truth.
3) **Scene 1:** Jane 'video calls' Derek who is about to board an aeroplane for work purposes. Jane expresses the need to consult with her GP because of chronic headaches (which are stress-related as a result of her abuse). However, Derek demands Jane doesn't seek an appointment with her GP until he returns home. Once the video call ends Jane really struggles with this dilemma and nervously rings her GP to schedule an appointment. Unexpectedly the GP receptionist offers Jane an immediate GP appointment (because of a cancellation). Normally Jane would only ever consult her GP in the presence of Derek. Jane nervously decides to accept this video consultation with her GP. While she waits to consult with her GP, she covers up the bruises on her neck with a scarf.
4) **Scene 2:** The GP video-consults with Jane. The GP focuses more on the biomedical aspects of her presentation (her tension headache). The GP does not pick up on the clues and cues from the patient that she may be DA. The consultation concludes with the GP providing medical advice regarding headaches. Underpinning this consultation were the following features portrayed by Jane:

a. Jane only ever attends her GP with Derek being present (due to his controlling nature);
b. She was concealing a bruise on her neck due to physical abuse inflicted by Derek;
c. Her tension headache was a manifestation of her DA;
d. She portrayed cues that she was nervous and uncomfortable during the consultation;
e. Jane really wants to leave Derek but felt that she couldn't do this due to his controlling nature. She really wants her GP to help her disclose and validate her suffering. In order to do this, her GP needs to be proactive in creating a supportive approach and asking her whether she is a victim of DA. On disclosure, the GP would enquire about safety issues and refer Jane patient on to a relevant agency.

5) **Scene 3:** In this scene, scene 2 is repeated. This time, however, participants ('spectactors') were invited to 'step into the shoes' of the GP and decide their actions; in effect as proxy actors. The actors that portrayed Jane and the GP reacted and improvised to the participants' directions - to the extent that the participants as 'spectactors' controlled the destiny of the consultation. This process was facilitated by GG who stopped the performance (or allowed participants to stop the performance) and discussed with the participants how to adjust the GP's actions to provide the best care for the patient. During these discussions, the actors switched off their video and muted their microphones.
6) **Debrief:** Participants were finally debriefed using the PEARLs model (Milan et al., 2006). Importantly, the actors engaged in the debrief and were introduced to the participants 'out of role'.
