# Supplementary material for: From ‘spectating’ to ‘spect-acting’: medical students’ lived experiences of online Forum Theatre training in consulting with domestic abuse victims
Source: Adv Simul (Lond). 2022 Apr 15;7:11. doi: 10.1186/s41077-022-00208-1 (PMC9012059; doi:10.1186/s41077-022-00208-1)
Supplement: Supplementary file 2 — Additional file 2. Interview Schedule. [file 41077_2022_208_MOESM2_ESM.pdf]

ADDITIONAL FILE 3: Interview Schedule

Medical students' lived experiences of online forum theatre in consulting with victims of domestic abuse.

Hello, my name is Daire McGrath – an intercalating medical student at QUB. Thank you for taking the time to participate in this study. During this interview you will be offered the opportunity to draw pictures and share your experience and opinions of the online forum theatre learning activity. I will ask questions to facilitate a conversation about your experience of this research topic. Please keep in mind that there are no "right" or "wrong" answers to any of the questions I will ask. If you do not wish to answer any of the questions during the interview, you may say so and we will move on. The purpose is to stimulate conversation and hear your opinion. I hope you will be comfortable speaking honestly and sharing your experience with me.

Please note that this session will be recorded to ensure I adequately capture your ideas during the conversation. However, the comments from the interview will remain confidential and your name will not be attached to any comments you make. It is anticipated the interview will last typically 45 minutes but can range from 30-60 minutes. You are free to stop the interview or withdraw at any time.

Do you have any questions before we begin? Can I now invite you to draw a picture that symbolises your experiences of the activity? Take the necessary time and material you require to create your drawing.

- Please share with me your experience of the forum theatre activity. What was your reaction? Tell me more? Why do you say that?
- How did it make you feel as a student? Why do you think it made you feel this way?
- How did it make you feel as a future doctor? Why do you think it made you feel this way?
- What was your experience of the acting? Did it feel realistic at times? Tell me more?
- Did this challenge your thinking? Tell me more?
- How did it feel to change the course of the acting by your discussions?
- What emotions where you feeling? Can you explain this to me?
- Was it being online less realistic? If so – tell me more?
- Has this experience made you reflect on your future role as a doctor? If so – can you share with me?
- How does this experience have any impact on you as a future doctor? Please tell me more

Thank you for your time and for sharing your experience with me. Do you have any questions?
